# Structure versus function: correlation between outer retinal and choroidal thicknesses measured by swept-source OCT with multifocal electroretinography and visual acuity

**DOI:** 10.1186/s40942-017-0082-y

**Published:** 2017-08-07

**Authors:** Ignacio Flores-Moreno, Luis Arias-Barquet, Marcos J. Rubio-Caso, Alex Muñoz-Blanco, María Vidal-Martí, Jaume Catala-Mora, José M. Ruiz-Moreno, Jay S. Duker, Josep M. Caminal

**Affiliations:** 1 Department of Ophthalmology, Bellvitge University Hospital, Feixa Llarga, s/n, 08907 L’Hospitalet de Llobregat, Barcelona Spain; 2 Department of Ophthalmology, Clínico San Carlos University Hospital, Madrid, Spain; 3 Department of Ophthalmology, Castilla La Mancha University, Albacete, Spain; 4 New England Eye Center, Tufts Medical Center, Boston, MA USA

**Keywords:** Choroidal thickness, Swept-source, OCT, Multifocal electroretinography, Visual acuity

## Abstract

**Background:**

To correlate retina-choroidal anatomy as assessed via swept-source OCT (SS-OCT) with retinal function as determined by best-corrected visual acuity (BCVA) and multifocal electroretinogram (mfERG).

**Methods:**

Thirty-three eyes from 33 patients including 16 with neovascular AMD (nvAMD) and 17 controls were included. Patients were included in the present study after a complete ophthalmologic examination, including BCVA, slit-lamp study, intraocular pressure measurement, dilated fundus examination after tropicamide instillation, SD-OCT, SS-OCT, fundus photographs and mfERG. Age, sex, BCVA, number of anti-VEGF intravitreal injections in the nvAMD group, were recollected. Outer retinal and choroidal thickness were determined at the fovea and 500 μm temporal, superior, nasal and inferior. First-order response from mfERG was collected. P1 amplitude was recorded in R1, R2 and the average of R1 + R2. The measurements recollected from the SS-OCT, mfERG and BCVA were compared.

**Results:**

Better BCVA was found with thicker outer retina foveal thickness (r = 0.349; *P* = 0.047), with thicker subfoveal choroidal thickness (r = 0.443; *P* = 0.010), and with higher amplitude in P1 at R1 (r = 0.346; *P* = 0.037). Outer retina foveal thickness did not correlate with P1 amplitude at R1 (r = 0.072; *P* = 0.692), R2 (r = 0.265; *P* = 0.137) either with the average P1 amplitude at R1 + R2 (r = 0.253; *P* = 0.156). A thicker subfoveal choroidal thickness was related with higher amplitude in P1 at R1 (r = 0.383; *P* = 0.028), R2 (r = 0.409; *P* = 0.018) and the average of R1 + R2 (r = 0.419; *P* = 0.015).

**Conclusions:**

Choroidal thickness demonstrated a positive correlation with retinal function in the sample studied, so a thicker choroid is related to a better retinal function measured with mfERG and BCVA.

## Background

The choroid plays an important role in the maintenance and proper functioning of the retina. The posterior part of the uvea supplies oxygen and nutrients to the outer retina in humans, and also to the inner retina in species with avascular retina [1]. The choroid serves as a thermoregulator dissipating the resulting heat of the retina metabolism [2], absorbs light and modulates the intraocular pressure via vasomotor control of blood flow and via the uveoscleral pathway [1].

Swept-source OCT (SS-OCT) allows excellent in vivo imaging of the choroid and sclera, as compared to time domain (TD) OCT or even spectral domain (SD) OCT [3, 4]. This technology employs a longer wavelength light source, around 1050 nm, compared to the 840 nm wavelength typically used in SD-OCT. Due to the higher imaging speed, more scans can be averaged in a shorter time frame resulting in higher quality scans. The uniform image quality over depth (no sensitivity roll off) appears to allow a better resolution in some chorioretinal diseases [5–7]. Multifocal electroretinogram (mfERG) is an electrophysiological test that provides objective information about retinal function. This test allows a topographic mapping of retinal function in the macula due to stimulation of different areas of the retina [8].

Recently, choroidal thickness has been correlated to visual acuity in a healthy population [2, 9]. A thicker choroid is related to a better visual acuity, in a population-based study, with patients older than 50 years old, independently of other factors as age, axial length, education level and major ocular diseases [9]. Visual acuity in high myopic patients has been associated to outer retina and choroidal thickness [10, 11]. The aim of this study is to correlate an anatomic test, SS-OCT, with functional tests, such as best-corrected visual acuity (BCVA) and mfERG, in two different groups: healthy controls and neovascular age-related macular degeneration (nvAMD).

## Methods

Thirty-three eyes from 33 patients were included in this observational, cross-sectional study. Two classes of patients were included: nvAMD and healthy control patients. One eye from each patient was included, in the nvAMD group, if both eyes were eligible, the one with better BCVA was the one included. In the control group, the right or left eye was enrolled in alternative fashion. Patients with nvAMD were selected for this study because it is well known that this disease show pathologic changes on mfERG [8, 12, 13]. Patients with spherical equivalent refraction of ±3 diopters were excluded in both groups, trying to make more homogeneous groups. Patients with media opacities were excluded. This study adhered to the tenets of the Declaration of Helsinki and was approved retrospectively by the Institutional Review Board (IRB)/Ethics Committee from Bellvitge University Hospital (Barcelona-Spain). Written inform consent was obtained in each patient included.

Patients were included in the present study after a complete ophthalmologic examination, including BCVA, slit-lamp study, intraocular pressure measurement, dilated fundus examination after tropicamide instillation, SD-OCT, SS-OCT, fundus photographs and mfERG. Age, sex, BCVA, number of anti-vascular endothelial growth factor (VEGF) intravitreal injections in the nvAMD group, were recollected.

The SS-OCT device employed was DRI-1 Atlantis OCT (Topcon Corporation, Tokyo, Japan) which uses a tunable laser as a light source operated at 100,000 Hz, A-scan repetition rate at 1050 nm of wavelength. The device can perform image averaging of up to 96 B scans at each location. For this study, the reference mirror was placed at a deeper position of the retina so that the sensitivity was higher at the choroid. Horizontal and vertical line scans containing 1024 axial scans with 12 mm length were performed centered in the fovea. Acquisition time was 1 s. To avoid circadian choroidal thickness changes, our patients were image between 10 p.m. and 2 p.m. [14].

**Fig. 1:**
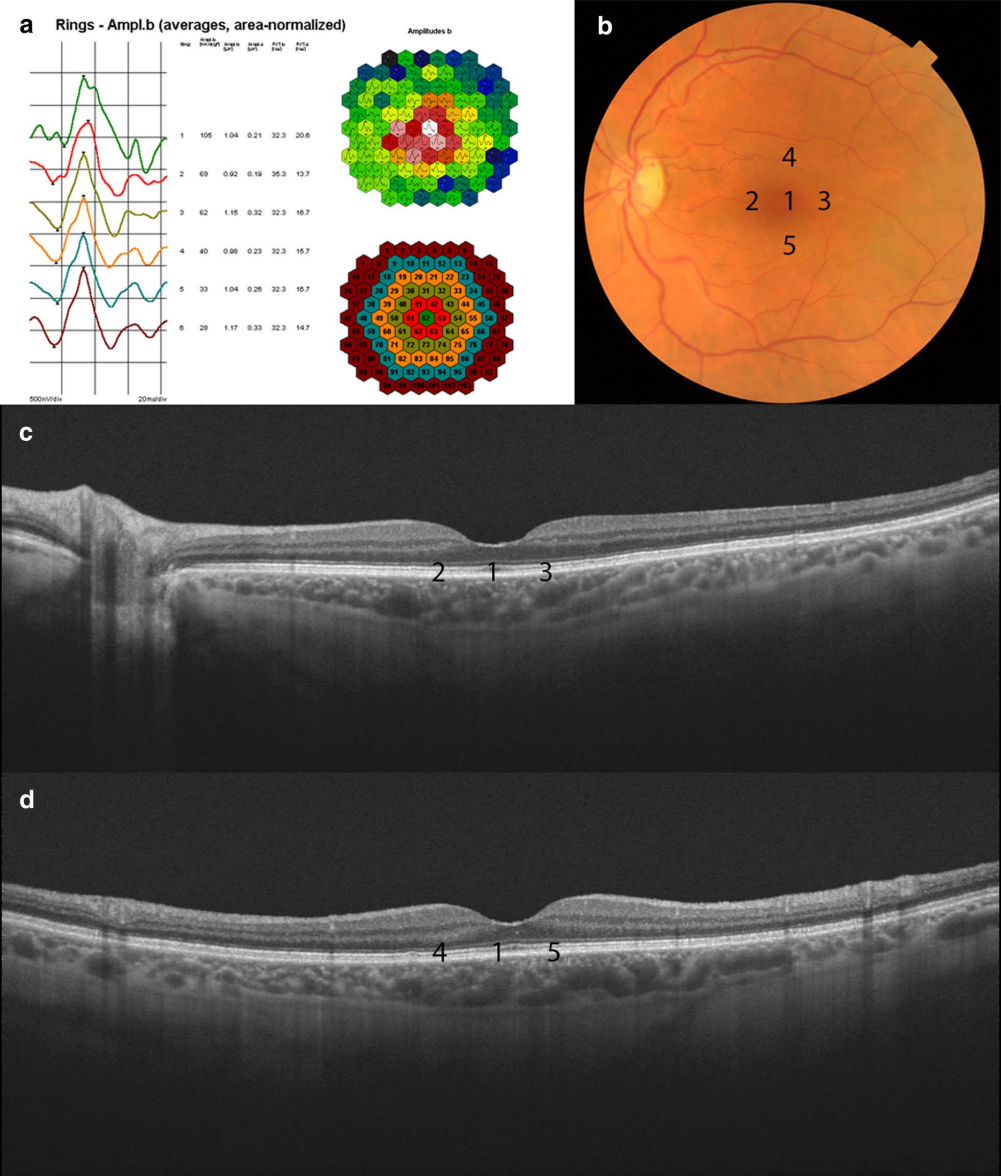
Multifocal electroretinogram (**a**), color fundus (**b**) and swept-source optical coherence tomography (SS-OCT) *horizontal* (**c**) and *vertical B-scan* (**d**) of a healthy control patient. The multifocal electroretinogram shows the diagrams of response from ring 1 to ring 6. *Color fundus* shows the places where the outer retina and choroidal measurements were made: fovea (*1*) and at 500 μm nasal (*2*), temporal (*3*), superior (*4*) and inferior (*5*) to the fovea. SS-OCT *horizontal* and *vertical B-scan* of swept-source showing the places where the measurements were taken

Two independent not-blinded investigators using the caliper system provided by the OCT system software manually performed the measurements. Outer retinal thickness was defined as the space between the hyperreflective band corresponding to the external limiting membrane and the outer border of the hyperreflective band corresponding with the retinal pigment epithelium (RPE), according to the recent description of anatomic layers in retina and choroid via OCT made by Staurenghi et al. [15]. Outer retinal measurements were made at the fovea and at 500 μm temporal, superior, nasal and inferior to the fovea. Choroidal thickness was defined as the space included between the outer border of the hyperreflective band corresponding with the RPE and the choroid scleral junction [15], at the subfoveal area and at 500 μm temporal, superior, nasal and inferior to the fovea (Fig. 1).

The mfERG test was performed using RetiScan/RetiPort (Roland Consult, Brandenburg, Germany) with software version 2.15, according to the International Society for Clinical Electrophysiology of Vision (ISCEV) guidelines [16]. The stimulus consisted of 103 hexagonal elements, which switched on and off according to a pseudorandom binary sequence. All patients were dilated with tropicamide and exposed to ordinary room lighting 15 min prior to test. Pupil dilatation was checked, and the test was performed under a constant dim light. Two consecutive mfERG measurements were made in each eye.

To compare the mfERG values with SS-OCT, first-order response was studied. P1 amplitude was recorded in ring (R) 1, R2 and the average of R1 + R2. These measurements inform about the foveal function, the parafoveal function and the central 5° of visual angle, respectively.

The measurements recollected from the SS-OCT, mfERG and BCVA were compared according to the corresponding examined area, so foveal and parafoveal (500 μm away from the fovea) were compared with R1, R2 and the average of R1 + R2 and with BCVA.

Statistics analysis was performed with SPSS software package version 20 (SPSS Inc, Chicago, IL, USA). Pearson correlation test was used to study the correlations between the SS-OCT, mfERG measurements and BCVA. Mann–Whitney *U* test was employed to compare groups. Intraclass correlation coefficient (ICC) was employed to measure the inter-observer agreement in the SS-OCT measurements. A *P* value of ≤0.05 was considered as statistically significant.

## Results

**Fig. 2:**
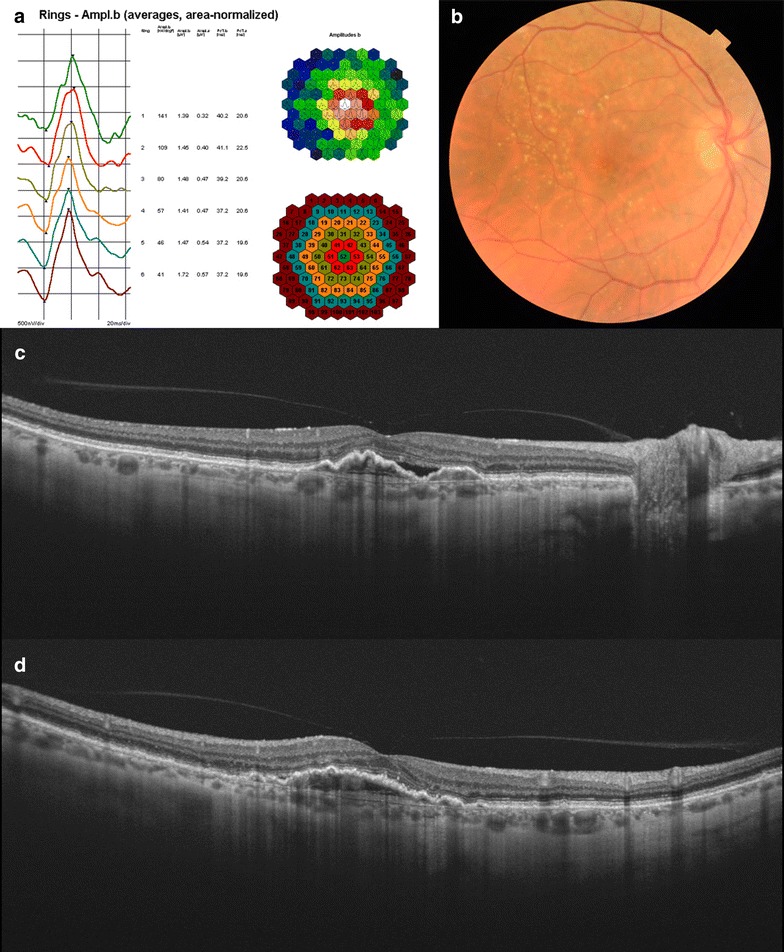
A 70-year-old female patient from the neovascular age-related macular degeneration group, who previously received 5 intravitreal injections of ranibizumab, with a best-corrected visual acuity of 0.4 (20/50). Multifocal electroretinogram (**a**), color fundus (**b**) and swept-source optical coherence tomography *horizontal* (**c**) and *vertical B-scans* (**d**) are shown. Similar *diagrams* are seen from R1 to R6, with high peak in P1 at the different rings. *Color fundus* shows hard drusen at the posterior pole, without fibrosis or scar. The *B-scans* show a focal vitreo-macular adhesion, with subretinal fluid and a retinal pigment epithelium detachment associated

The study enrolled 33 eyes from 33 patients. Sixteen patients from the nvAMD group (Fig. 2), and seventeen from the control group. 69% were female, with a mean age of 62.8 years old ± 14.5 (from 33 to 86). Mean visual acuity from the entire sample was 0.68 ± 0.3 (from 0.05 to 1; from 20/400 to 20/20), mean visual acuity in the AMD group was 0.38 ± 0.32 (from 0.05 to 0.9), mean visual acuity in the control group was 1 ± 0.0 (from 1 to 1). All the patients from the nvAMD group received intravitreal anti-VEGF injections, with a mean of 6.9 injections ± 4.5 (from 2 to 20). The rest of patients included did not receive previous ophthalmic treatment.

Mean outer retina thickness of all the patients studied at the fovea was 108.1 μm ± 30.9 (from 69 to 242), mean outer retina thickness 500 μm away from the fovea was 86.6 μm ± 32.8 (from 20 to 147). Mean subfoveal choroidal thickness was 241.1 μm ± 93.1 (from 101 to 493), mean choroidal thickness 500 μm away from the fovea was 234.5 μm ± 86.2 (from 111 to 411). Mean P1 amplitude at R1 was 106.4 nanoV/deg^2^ ± 52.3 (from 38.4 to 260), at R2 was 72.4 nanoV/deg^2^ ± 26.1 (from 34 to 148) and mean average of R1 + R2 was 73.7 nanoV/deg^2^ ± 26.8 (from 38.6 to 149). Characteristics of each group are summarized in Table 1.

**Table 1:** Mean swept-source optical coherence tomography and multifocal electroretinography measurements from the two groups of patients

	NvAMD (mean ± SD)	Control (mean ± SD)	*P* value (Mann–Whitney *U* test)
Mean outer retina thickness at the fovea (µm)	107.5 ± 64.1	102.3 ± 6.2	0.001*
Mean outer retina thickness at 500 μm	78.6 ± 34.0	88.6 ± 5.5	0.001*
Mean subfoveal choroidal thickness (μm)	226.1 ± 95.1	325.4 ± 101.9	0.001*
Mean choroidal thickness at 500 μm	222.7 ± 85.3	328.6 ± 99.9	0.001*
Mean P1 amplitude at R1 (nV)	90.7 ± 37.3	121.2 ± 60.8	0.150
Mean P1 amplitude at R2 (nV)	63.6 ± 20.1	80.8 ± 28.8	0.105
Mean P1 average of R1 + R2 (nV)	64.8 ± 20.8	82.2 ± 29.7	0.130

*nvAMD* neovascular age-related macular degeneration, *SD* standard deviation

* Statistical significance

Simple regression analysis showed statistical significance in some of the studied parameters. Better BCVA was found in thicker outer retina foveal thickness (r = 0.349; *P* = 0.047), and with thicker outer retina at 500 μm away from the fovea (r = 0.662; *P* < 0.001), also a better BCVA with thicker subfoveal choroidal thickness (r = 0.443; *P* = 0.010), and thicker choroidal thickness at 500 μm away from the fovea (r = 0.466; *P* = 0.006), and with higher amplitude in P1 in the central parameters of mfERG (P1 value at R1 (r = 0.346; *P* = 0.037), R2 (r = 0.374; *P* = 0.032) and the average of R1 + R2 (r = 0.375; *P* = 0.032). Outer retina foveal thickness did not correlate with P1 amplitude at R1 (r = 0.072; *P* = 0.692), R2 (r = 0.265; *P* = 0.137) either with the average P1 amplitude at R1 + R2 (r = 0.253; *P* = 0.156). Thicker outer retina thickness at 500 μm away from the fovea was related to a thicker choroidal thickness at 500 μm away from the fovea (r = 0.478; *P* = 0.005) and with a higher amplitude in P1 value at R2 (r = 0.353; *P* = 0.044). Thicker subfoveal choroidal thickness was found in higher amplitude of P1 at R1 (r = 0.383; *P* = 0.028), R2 (r = 0.409; *P* = 0.018) and the average of R1 + R2 (r = 0.419; *P* = 0.015). Thicker choroidal thickness 500 μm away from the fovea showed correlation with higher amplitude of P1 at R2 (r = 0.369; *P* = 0.034), and the average of R1 + R2 (r = 0.371; *P* = 0.033). Higher amplitude of P1 at R1 showed a correlation with better BCVA (r = 0.346; *P* = 0.037) and with thicker subfoveal choroidal thickness (r = 0.383; *P* = 0.028), not with outer retina foveal thickness (r = 0.072; *P* = 0.692) (Table 2).

**Table 2:** Correlation between best corrected visual acuity (BCVA), foveal and parafoveal outer retinal thickness, subfoveal and parafoveal choroidal thickness and P1 valor at R1, R2 and the average of R1 + R2

	BCVA	Foveal RT	500 μm RT	Subfoveal CT	500 μm CT	R1	R2
Foveal RT	0.349 *P* < 0.047*						
500 μm RT	0.662 *P* < 0.001*	0.873 *P* < 0.0001*					
Subfoveal CT	0.443 *P* = 0.010*	0.355 *P* = 0.157	0.273 *P* = 0.060				
500 μm CT	0.466 *P* = 0.006*	0.289 *P* = 0.204	0.478 *P* = 0.005*	0.963 *P* < 0.001*			
R1	0.346 *P* = 0.037*	0.072 *P* = 0.692	0.126 *P* = 0.473	0.383 *P* = 0.028*	0.285 *P* = 0.063		
R2	0.374 *P* = 0.032*	0.265 *P* = 0.137	0.353 *P* = 0.044*	0.409 *P* = 0.018*	0.369 *P* = 0.034*	0.780 *P* < 0.001*	
R1 + R2	0.375 *P* < 0.032*	0.253 *P* = 0.156	0.395 *P* = 0.006*	0.419 *P* = 0.015*	0.371 *P* = 0.033*	0.823 *P* < 0.001*	0.996 *P* < 0.001*

Pearson correlation test was performed. A *P* value of ≤0.05 was considered as statistically significant

*BCVA* best-corrected visual acuity, *RT* retinal thickness, *CT* choroidal thickness

* Statistical significance

No correlation was found between parameters in each group separately, probably due to the low number of patients included in every group.

To compare the manual measurements performed by the two investigators, ICC was employed. Inter-observer correlation was 0.792 for the outer retina measurements and 0.962 for the choroidal measurements. These values are considered substantial (0.61–0.80) and almost perfect (0.81–1) in the classification of Landis and Koch [17].

## Discussion

The practice of ophthalmology has been revolutionized by recent improvements in ocular imaging, in particular OCT. The development of enhanced depth imaging (EDI) SD-OCT allowed the visualization of deeper structures in the posterior pole, displaying the choroid adjacent to the zero delay line [18]. In contrast, SS-OCT uses a longer wavelength light source and can perform average of higher number of scans over a similar time frame [19], both of which improve the quality of OCT images when studying the choroid and sclera.

Because of the ability of SD-OCT to image the choroid, its study has become routine for many retinal practitioners, thereby facilitating the understanding of the pathophysiology of certain diseases and assisting the diagnosis of other pathologies. For example, a thicker subfoveal choroidal thickness at initial stages of diabetic macular edema has been related to a better anatomic and functional outcome in patients treated with anti-VEGF therapy [20]. Kang et al. [21] demonstrated that patients, with greater subfoveal choroidal thickness affected by nvAMD showed a better response to anti-VEGF and correlated with better final BCVA, after 6 months of follow-up. These authors proposed that a thicker choroid preserves the choriocapillaris and is correlated with better choroidal blood circulation, which will in the end determine a better visual acuity [20, 21].

Visual acuity and outer foveal thickness have been positively correlated in high myopia [10] and also with choroidal thickness in a large population-based study [9] as well as in high myopic patients without macular pathology [10, 11]. Thinner choroids are associated with worse visual acuity, as confirmed in the present study, where BCVA is correlated to foveal and perifoveal outer retinal thickness and choroidal thickness. In this study, better BCVA is associated with a more robust mfERG foveal and perifoveal response.

Choroidal thickness has been found to correlate with retinal function measured by mfERG in the foveal and perifoveal area. The choroid supplies oxygen and nutrients to the outer retina, the choroid acts as a heat dissipater from the retinal metabolism, allowing a better function of the photoreceptors [1]. So a thicker choroid may imply an improved photoreceptors metabolism, a better oxygenation of the outer retina a better visual acuity and better function, as it has been demonstrated in this study using P1 amplitude from mfERG.

Palmoski–Wolfe suggested that morphometric tests, such as OCT, can not replace functional retinal tests, due to failure to find a strong correlation between OCT and mfERG in eyes with pathology [22]. SS-OCT cannot replace functional tests, as mfERG, but this study demonstrates that the development of new technology in imaging, and higher resolution in our images, can complement functional tests, that are more time-consuming and the correlation between the function and anatomical changes at outer retina and choroid has been probed.

This study has several limitations including the relatively low number of patients included in each group. SS-OCT measurements were manually performed, due to the fact that there is no commercially available automated software available. No correction was made for other variables such as central corneal thickness, intraocular pressure, systemic medication and blood pressure. Pathologies with abnormally thick choroid, as Vogt–Koyanagi–Harada [23], central serous chorioretinopathy [18] or polypoidal choroidal vasculopathy [21], have not been included in the present study, so the results cannot be extensive to these conditions with thicker choroid, other ways high myopic patients without macular pathology have been proofed to have thinner choroid and a correlation between BCVA and outer retina and choroidal thickness [10, 24], as it happens in this study.

## Conclusion

To conclude, choroidal thickness, determined with a non-invasive, rapid, high-resolution technology, as SS-OCT, have been demonstrated to have a positive correlation with retinal function, measured with mfERG and with BCVA. Future prospective studies with higher number of patients need to be performed to corroborate these findings.

## Abbreviations

SS-OCT: swept-source OCT
BCVA: best-corrected visual acuity
mfERG: multifocal electroretinogram
nvAMD: neovascular age-related macular degeneration
TD: time-domain
SD: spectral-domain
RPE: retinal pigment epithelium
ICC: intraclass correlation coefficient
EDI: enhanced depth imaging
